# Knee replacement surgery in a patient with acquired von Willebrand disease: a case study with recommendations for patient management

**DOI:** 10.1097/MS9.0000000000001690

**Published:** 2024-01-08

**Authors:** María Teresa Álvarez Román, María Isabel Rivas Pollmar, Hortensia De la Corte-Rodríguez, Primitivo Gómez-Cardero, E. Carlos Rodríguez-Merchán, Mar Gutiérrez-Alvariño, Eduardo García-Pérez, Mónica Martín-Salces, Damaris Zagrean, Nora V. Butta-Coll, Víctor Jiménez-Yuste

**Affiliations:** Departments ofa Hematology; b Physical and Rehabilitation Medicine; c Orthopedic Surgery, La Paz University Hospital, IdiPaz; d Osteoarticular Surgery Research, Hospital La Paz Institute for Health Research, IdiPAZ (La Paz University Hospital—Autonomous University of Madrid); e Department of Medicine, Autonomous University of Madrid, Madrid, Spain

**Keywords:** bleeding, orthopaedic, surgery, Von Willebrand factor, Willfact®

## Abstract

**Introduction and importance:**

Acquired von Willebrand disease (AvWD) is a rare underdiagnosed bleeding disorder caused by alterations in the levels of the major blood-clotting protein von Willebrand factor (vWF). The clinical and laboratory parameters of AvWD are similar to congenital vWD, but it is found in individuals with no positive family history with no underlying genetic basis. The disease remains multifactorial and incompletely understood. Proposed mechanisms include the development of autoantibodies to vWF, absorption of high molecular weight vWF multimers that impair normal function, shear stress induced vWF cleavage and increased proteolysis.

The aetiology of the disease is variable, the most common being hematoproliferation, lymophoproliferation, myeloproliferation and autoimmune and cardiovascular disorders. Consensus and protocols for AvWD patients that require major surgery are currently lacking. Patients with AvWD can experience thrombotic events during surgery as a result of therapeutic interactions with pro-thrombotic risk factors.

**Case presentation:**

Here, the authors report a patient with AvWD requiring a knee prosthesis implantation due to chronic pain, limited range of motion and functional impairment. The patient had a high risk of bleeding during surgery and was at risk of thrombosis due to age and obesity.

**Clinical discussion:**

Perioperative care required a collaborative approach and the management of bleeding. The patient was administered vWF concentrate Willfact lacking Factor VIII to prevent haemorrhage and to minimize the risk of thrombosis.

**Conclusion:**

The treatment was effective and well-tolerated. The authors use this information to provide recommendations for AvWD patients for whom major surgery is indicated.

## Introduction

Highlights

- Acquired von Willebrand disease is a rare disease.
- Information about the management of these patients is essential.
- The clinical case presented here is important because it is a patient with a high risk of bleeding and high thrombotic risk due to surgery and pre-existing comorbidities.
- The use of pure VWF concentrates that do not increase the thrombotic risk in these patients is essential.
- Experience in this pathology is scarce, as is the management of orthopaedic surgery in a patient with comorbidities and treated with a pure VWF such as Wilfactin.

von Willebrand factor (vWF) is a multimeric glycoprotein that plays a key role during haemostasis following vascular injury^1^. vWF promotes coagulation by acting as a carrier for the essential blood-clotting protein factor VIII (FVIII), preventing its degradation by activated protein C^2^. vWF is released from injured endothelial cells and is an important biomarker of endothelial dysfunction^3^ vWF promotes platelet adhesion at the sites of vascular damage, mediating thrombus formation through interactions with collagen and platelet receptors Ib, IX and V^4^. Deficiencies of vWF (≤50 IU/dl) result in the bleeding disorder Von Willebrand disease (vWD)^5^.

vWD can be inherited (autosomal dominant) or acquired^6^. Acquired deficiency or dysfunction of vWF (AvWD) is similar to congenital vWD regarding clinical and laboratory parameters, but is found in individuals with no positive family history. AvWD is less common than genetic vWD and diagnosis is often not suspected in a bleeding patient^7^.

The development of AvWD is multifactorial. Common causes include the presence of autoantibodies that increase the plasma clearance of vWF and the absorption of high molecular weight and functionally impaired vWF multimers^8^. Waldenstrom macroglobulinemia promotes selective vWF immuno-adsorption due to lymphocyte dysfunction^9^. A similar mechanism was reported in a patient with multiple myeloma^10^. Increased proteolytic cleavage of vWF can occur as a result of intravascular shear stress during aortic stenosis, leukaemia and pancreatitis^11–13^. Impaired vWF production due to hypothyroidism and drugs that influence haemostatic parameters are also associated with AvWD development^14,15^. Individuals with AvWD manifest excessive mucocutaneous bleeding, bruising, epistaxis and menorrhagia.

A major challenge during the management of AvWD patients is the high risk of bleeding during major surgery. Consensus on the best practice for treatment aims and postoperative tests are lacking^16–19^. Recent guidelines on inherited vWD recommend the use of Willfact (human VWF) for the prevention of haemorrhages or surgical bleeding when desmopressin is ineffective or contraindicated^20^. Whilst Willfact is commonly used to support clotting in AvWD patients, its use during surgery has not been documented.

Here, we report the treatment of an elderly female patient with AvWD undergoing major knee surgery. We provide evidence-based guidelines for the management of AvWD during surgery and the use of vWF concentrates in this patient cohort.

## Methods

### Case presentation

A 71-year-old woman with suspected AvWD was referred to our clinic for the first time in February, 2010 and treated from 2010 to 2022. Her past medical history included a normal pregnancy at 24 years of age, mastectomy, radiotherapy and chemotherapy for carcinoma of the left breast at 38 years, lacrimal duct agenesis surgery at 51 years, and meniscus tear surgery at 67 years of age. No complications or bleeding were associated with any of these treatments. The patient was in complete remission from the carcinoma. The patient was diagnosed with advanced arthrosis in the right knee and hip aged 60. Cardiovascular risk factors including dyslipidaemia, hypertension, hyperglycaemia and obesity were recorded. A family history of bleeding was noted.

Significant history included the development of frequent epistaxis and bleeding following a dental extraction at the age of 60 years. The patient was treated at our clinic over a period of 12 years (2010–2022) for retinal haemorrhage; recurrent hemarthrosis; and advanced gonarthrosis. The patient had poorly controlled pain and required treatment with oxycodone hydrochloride, paracetamol, metamizole, and infiltrations with triamcinolone 40 mg and ropivacaine (0.1% at a total dose of 8 ml) every 6–8 months. This case report follows the guidance of SCARE (Surgical Case Report) criteria^21^.

### Diagnosis

**Figure 1:**
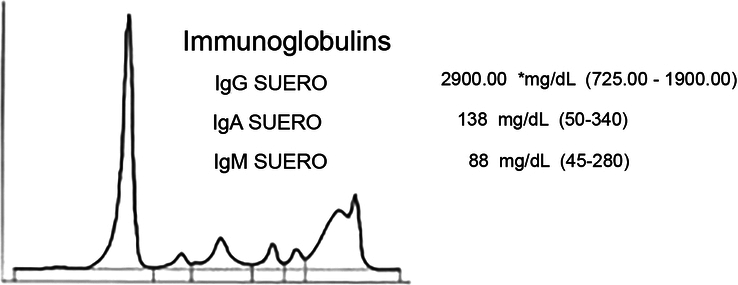
Serum electrophoresis. Peaks associated with monoclonal gammopathy of undetermined significance.

**Figure 2:**
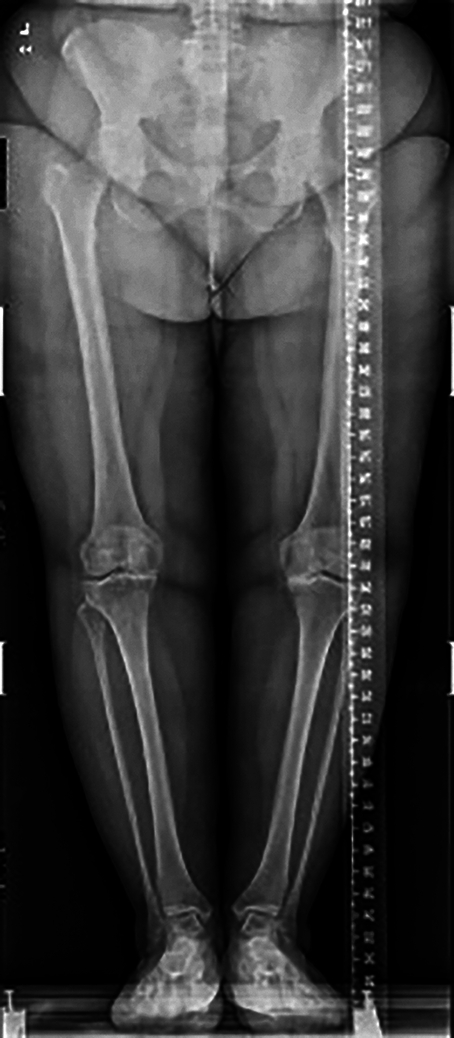
Plain films revealing varus deformities and osteoarthritis in both knees.

In 2012, the patient was diagnosed with AvWD associated with Monoclonal Gammopathy of Undetermined Significance (MGUS) (Fig. 1). The diagnosis of AvWD was made based on a negative family history of bleeding, recent onset bleeding, elongated aTTP, and the frequent association of AvWD with MGUS^22–24^. In 2022, the patient was evaluated for chronic pain in the knees with functional impairment and limited mobility (range of motion: 0–90^o^). Imaging studies revealed arthropathy that led to varus deformities of both knees (Fig. 2). The patient had a body mass index of 30 and did not perform physical activity. A right knee prosthesis implantation was recommended. The patient was considered at a high risk of bleeding and thrombosis. The patient had a partial response to DDAVP which was deemed not suitable for the implantation of a knee prosthesis as haemostatic levels must be maintained for extended time periods and tachyphylaxis can occur after 48 h. Patient management was planned by a team of orthopaedic, haematology, pharmacy and rehabilitation medicine specialists. The patient was diagnosed with the IgG MGUS shortly after diagnosis due to elevated serum proteins. Imunofixation analyses detected monoclonal IgG k.

## Results

### Pre-surgical management

Routine preoperative laboratory findings were: platelet counts: 403 000/l (normal: 150–400×10^9^/l), Hb: 8.9 g/dl (12–15.6), MCV: 70 fl (80–99), and Iron profiles: Fe: 13 (50–170) mcg/dl; Ferritin: 6 ng/ml (12–150 ng/ml). Coagulation studies were Fibrinogen: 4.75 g/l (1.5–4.5 g/l), FVIII:C:14%, VWF:Ag: 9.8%, VWF:RCo: 6.8%, FXI:C: 101.8% and FXII:C: 120.3%.

The preoperative levels of both VWF and FVIII were less than or equal to 10% of normal values. Preoperative factor tests were performed and the patient was administered a single dose of human recombinant FVIII (rFVIII) (20 IU/Kg) and Willfact (50 IU/Kg) in combination. Willfact was not administered alone as the half-life of the infused concentrates are shorter in AVWD patients. Pure Willfact was selected to reduce FVIII accumulation and thus the thrombotic risk. Single dose tests showed high recovery of FVIII and VWF, albeit with a shorter half-life than normal (Table 1).

**Table 1:** Blood parameters 2 weeks prior to surgery

Time (hours after factor administration)	FVIII:C (normal range 100) (%)	VWF: RCo (normal range 100) (%)
Basal level	9.2	6
30 min	60.5	84.3
60 min	41.5	35.3
6 h	32	10.2
11 h	24.7	5.2

FVIII: C, factor VIII activity; VWF: RCo, von Willebrand Ristocetin cofactor.

Table 2 shows the FVIII/ vWF: RCo levels during surgery and postoperatively. On the day of surgery, a single dose of rFVIII 20 UI/kg was administered with Willfact (50 IU/Kg). Maintenance doses of Willfact (50 IU/Kg) were administered every 8 h for the first 4 days and every 12 h from days 5 to 8. Due to difficulties in maintaining adequate levels of FVIII and VWF, no anticoagulant treatments were used during surgery.

**Table 2:** FVIII/ vWF: RCo levels during and after surgery

	Day of surgery	Day+1	Day+2	Day+4	Day+6	Day+7	Day+8
Basal levels	FVIII: C:6.6% VWF: RCo:3.5% Hb: 12.7 g/dl	FVIII:C:35.3% VWF:RCo:11.1% Hb: 10.3 g/dl	FVIII:C:43% VWF: RCo:7.2% Hb: 9.6 g/dl	FVIII: C:47.2% VWF: RCo:5.4% Hb:9.8 g/dl	FVIII: C:37.2% VWF: RCo:5.4% HB. 9.9 g/dl	FVIII:C:32.5% VWF: RCo:4.2% Hb: 9.5 g/dl	FVIII:C:34.5%; VWF:RCo: 5.4% Hb: 9.9 g/dl
	rFVIII 20 IU/Kg pdFVW 50 IU/Kg	pdFVW 50 IU/Kg	pdFVW 50 IU/Kg	pdFVW 50 IU/Kg	pdFVW 50 IU/Kg	pdFVW 50 IU/Kg	pdFVW 50 IU/Kg
Cmax	FVIII:C:58.6% VWF: RCo:54.8%	FVIII:C:49.6% VWF: RCo:54.9%	FVIII:C:42% VWF: RCo:50%	FVIII: C:49.3% VWF: RCo:58%	FVIII:C: 43.4% VWF:RCo 56.4%	FVIII:C:34.5% VWF: RCo:28.1%	FVIII:C: 34.8% VWF:CR:21.3%
8 h post-surgery	FVIII:C:74.2% VWF:RCo:36.8%	NR	NR	NR	NR	NR	NR
	pdFVW 50 IU/Kg	pdFVW 50 IU/Kg	pdFVW 50 IU/Kg	pdFVW 50 IU/Kg	NR	NR	NR
12 h post-surgery	FVIII: C:35.2% VWF: RCo:8.5%	NR	NR	NR	NR	NR	NR
	pdFVW 50 IU/Kg	pdFVW 50 IU/Kg	pdFVW 50 IU/Kg	pdFVW 50 IU/Kg	pdFVW 50 IU/Kg	pdFVW 50 IU/Kg	pdFVW 50 IU/Kg

Cmax, maximum concentration; FVIII: C, factor VIII activity; Hb, heamoglobin; NR, not reported; VWF: RCo; von Willebrand Ristocetin cofactor.

### Surgical procedure

**Figure 3:**
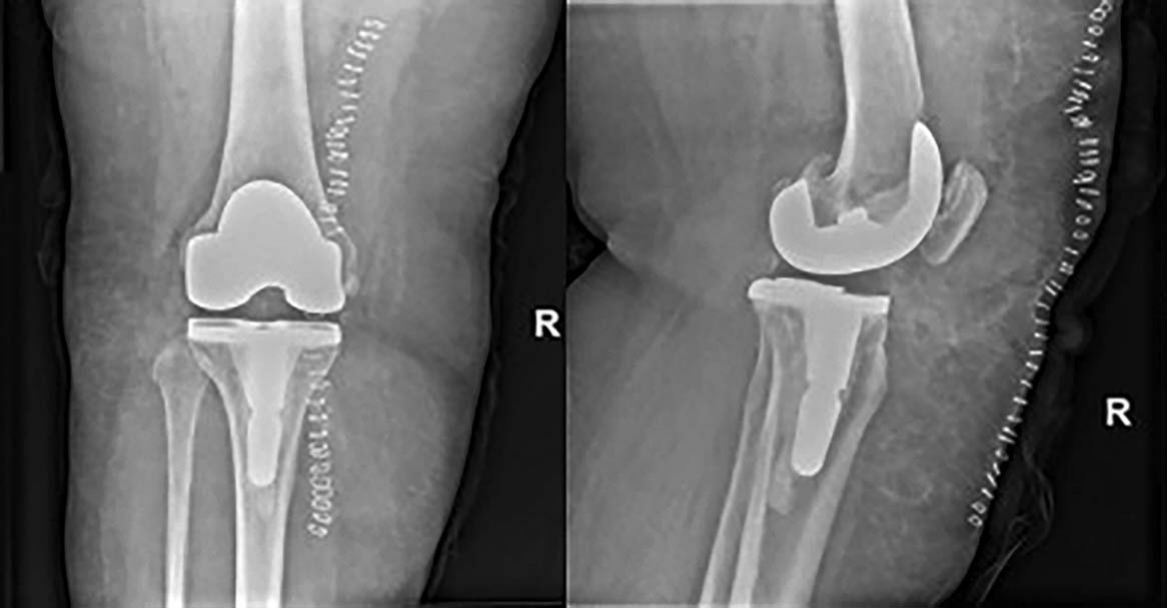
Post-surgical films showing successful implantation of the knee replacement.

Following successful implantation (Fig. 3), coagulation was managed through blood loss through the drains, blood haemoglobin and FVIII levels (Table 2). The patient showed no signs of a coagulation, thrombotic events or FVIII accumulation. The patient did not meet the criteria for corticosteroid therapy.

### Post-surgical management

Anticoagulants were administered early after surgery to reduce the risk of thrombosis. In patients with AvWD, the VWF concentrate is rapidly eliminated and does not protect from the risk of bleeding. The patient began mobilization and movement exercises within 24 h of surgery.

### Postoperative outcome

**Figure 4:**
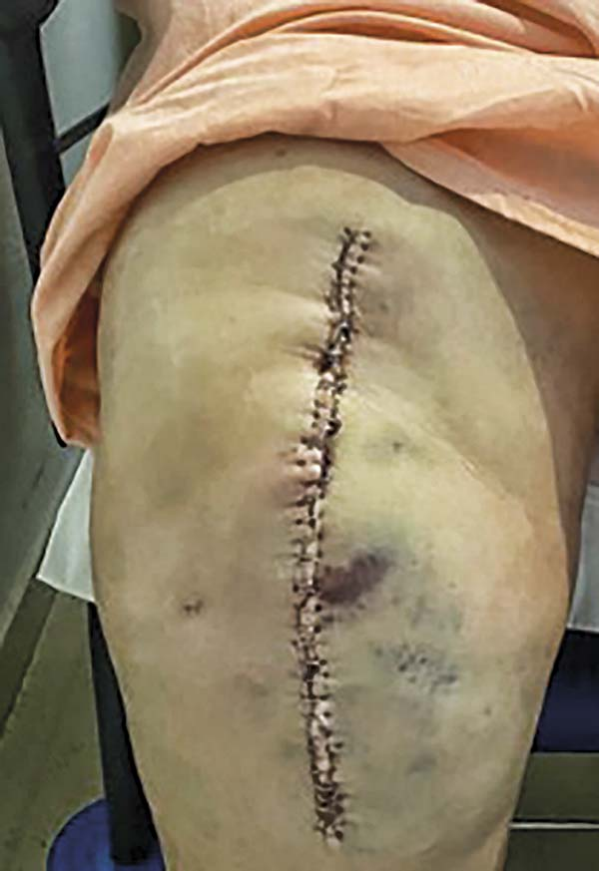
Knee swelling after the operation.

The day after surgery, the patient was in good condition, was afebrile, had well-controlled pain and no bleeding or deep vein thrombosis. Six days post-surgery, the patient developed knee pain, which was particularly intense in the popliteal fossa, both at rest and when walking. The extremities showed significant swelling and intense pain on examination (Fig. 4). Knee swelling, pitting oedema, skin erythema and a subcutaneous haematoma occurred. A Homan’s sign test could not be performed. Laboratory tests included Cmax FVIII: C: 43.4% and VWF: RCo 56.4%. A musculoskeletal ultrasound was performed, showing patent popliteal vessels and minimal joint fluid. The episode resolved spontaneously with the patient reporting improved mobility, increased physical activity and decreased pain.

## Discussion

The perioperative management of AvWD is challenging due to unpredictable coagulation pharmacokinetics and an increased risk of bleeding and thrombosis^16,17^. Patients with AVWD show defective VWF production but normal FVIII levels^25–29^. The administration of commercially available FVIII concentrates are required following major orthopaedic surgery which can increase the risk of thrombosis^30^. This risk is further increased by age, obesity and cardiovascular disease.

Desmopressin and pdFVIII/vWF concentrates form the cornerstone of VWD management. Desmopressin is effective in individuals with mild to moderately severe vWD with FVIII and vWF levels above 10% (type 1 and type 2A). pdvWF/FVIII concentrates can control and prevent bleeding in patients with more severe vWF and FVIII deficiency (type 3)^24^.

In this study, perioperative care required a collaborative approach and the management of bleeding parameters. VWF concentrate Willfact lacking Factor VIII was used to prevent haemorrhage and surgical bleeding and to minimize the risk of thrombosis. The treatment was effective and well-tolerated.

A single dose of vWF requires 6–12 h to increase FVIII: C concentrations to therapeutic levels. When haemostasis requires immediate correction during haemorrhage, severe trauma or emergency surgery, FVIII products must be administered with the initial vWF injection to ensure basal plasma levels. Table 2 illustrates how FVIII/ vWF: RCo levels were effectively maintained in the absence of FVIII accumulation in our patient (normal range: 50–200 IU/dl).

The replacement therapy of choice during the perioperative period is dependent on baseline FVIII activity, the Von Willebrand Ristocetin Cofactor range, the pattern of reaction to desmopressin dosage and the type of operation^31^. The use of desmopressin alone with prolonged periods of replacement therapy is limited by the increased incidence of tachyphylaxis after 48 h^32^. Replacement therapy containing FVIII and vWF can increase the risk of thrombosis. When baseline levels of FVIII are low, dual vWF/ FVIII products are effective. In patients with normal FVIII levels, dual administration can induce thromboembolism^33^. To avoid rapid increases in FVIII levels, high-purity vWF concentrates have been developed which are of low Factor VIII content (Willfact, LFB-Biomedicaments).

Willfact therapy should be initiated at 12–24 h prior to surgery and 1 h prior to elective surgery. Co-administration of FVIII was not necessary in our patient as endogenous FVIII achieved threshold levels of 0.4 IU/ml (40%). This should however be monitored on a patient-by-patient basis^21^. Surprisingly, the multimeric pattern was type 3. Genetic studies showed no pathogenic variants.

Close monitoring of bleeding parameters during and after surgery are necessary. The use of a concentrate that does enhance the accumulation of FVIII is also essential. Pure VWF concentrates such as Willfact, contain minimal FVIII, which minimizes the risk of thrombosis^34^.

### Recommendations for AvWD patients undergoing surgery

vWF, hepatic status and renal function should be characterized prior to surgery. Surgery should be performed in centres experienced in the management of haemorrhagic coagulopathies with freely available laboratory assays for Factor VIII vWF, Ag, and Von Willebrand Ristocetin Cofactor [vWF: RCo]).

Close monitoring of bleeding parameters during surgery is necessary. The vWF concentrate Willfact contains low levels of FVIII and data from this case report supports its efficacy and safety during major surgery.

We recommend that joint consultations are regularly performed amongst the departments of Orthopaedic Surgery, Haematology, Rehabilitation, Pharmacy and Nursing for patients with bleeding disorders requiring major surgery. The orthopaedic surgeon should present the surgical technique and allow the haematologist to design the haemostatic support. Rehabilitation physicians should ensure that the patient arrives at the surgery in the best possible condition. An individualized post-surgical rehabilitation program should be designed to address pain control, joint function recovery and gait retraining. The physiotherapist should initiate rehabilitation within 24 h of surgery.

## Ethics approval and consent to participate

Not applicable.

## Consent for publication

All authors read the manuscript and gave their final approval for publication.

## Sources of funding

This research received no specific grant from any funding agency in the public, commercial, or not-for-profit sectors.

## Author contribution

M.T.A.R. have designed the work. All the authors were responsible for the patient’s surgery, treatment and follow-up and have revised and gave final approval of the manuscript.

## Conflicts of interest disclosure

M.T.Á.-R. has participated as a speaker in advisory boards and sponsored symposia with Novo Nordisk, Bayer, Takeda, Roche, Pfizer, Octapharma, Amgen, Novartis, CSL Behring and Sobi. H.d.l.C.-R. has received honoraria for attending symposia/congresses and/or for speaking and/or consulting, and/or funds for research from Pfizer, Roche, Sobi, Novo Nordisk, Takeda and Bayer. V.J.Y. has participated as a speaker in advisory boards and sponsored symposia with Novo Nordisk, Bayer, Takeda, Roche, Pfizer, Octapharma, Amgen, Novartis, CSL Behring and Sobi. The remaining authors declare no conflict of interest.

## Research registration unique identifying number (UIN)

No applicable.

## Guarantor

The data that support the findings of this study are available from the corresponding author upon reasonable request.

## Availability of data and materials

The datasets used and/or analyzed during the current study are available from the corresponding author on reasonable request.

## Provenance and peer review

No applicable.

## References

[1] Harris NS Pelletier JP Marin MJ. Von Willebrand factor and disease: a review for laboratory professionals. Crit Rev Clin Lab Sci 2022;59:241–256. 34962443 10.1080/10408363.2021.2014781

[2] De Pablo-Moreno JA Serrano LJ Revuelta L. The vascular endothelium and coagulation: homeostasis, disease, and treatment, with a focus on the von Willebrand factor and factors VIII and V. Int J Mol Sci 2022;23:8283. 35955419 10.3390/ijms23158283 PMC9425441

[3] Denis CV Lenting PJ. How to keep the factor VIII/von Willebrand factor complex in the circulation. Haematologica 2022;107:2011–2013. 34818875 10.3324/haematol.2021.280222 PMC9425298

[4] Ruggeri ZM. Structure of von Willebrand factor and its function in platelet adhesion and thrombus formation. Best Pract Res Clin Haematol 2001;14:257–279. 11686099 10.1053/beha.2001.0133

[5] Kalot MA Husainat N El Alayli A. von Willebrand factor levels in the diagnosis of von Willebrand disease: a systematic review and meta-analysis. Blood Adv 2022;6:62–71. 34610118 10.1182/bloodadvances.2021005430 PMC8753202

[6] Casonato A Galletta E Cella G. Acquired von Willebrand syndrome hiding inherited von Willebrand disease can explain severe bleeding in patients with aortic stenosis. Arterioscler Thromb Vasc Biol 2020;40:2187–2194. 32640909 10.1161/ATVBAHA.120.314656

[7] Petricevic M Knezevic J Samoukovic G. Diagnosis and management of acquired von Willebrand disease in heart disease: a review of the literature. Thorac Cardiovasc Surg 2020;68:200–211. 30458570 10.1055/s-0038-1673670

[8] Nichols WL Rick ME Ortel TL. Clinical and laboratory diagnosis of von Willebrand disease: a synopsis of the 2008 NHLBI/NIH guidelines. Am J Hematol 2009;84:366–370. 19415721 10.1002/ajh.21405 PMC5592788

[9] Budde U Schaefer G Mueller N. Acquired von Willebrand’s disease in the myeloproliferative syndrome. Blood 1984;64:981–985. 6333259

[10] Richard C Cuadrado MA Prieto M. Acquired von Willebrand disease in multiple myeloma secondary to absorption of von Willebrand factor by plasma cells. Am J Hematol 1990;35:114–117. 2205095 10.1002/ajh.2830350210

[11] Tanaka H Nagai Y Kuwabara C. Acquired von Willebrand syndrome due to aortic valve stenosis in a case with antiphospholipid antibody. Intern Med 2018;57:1641–1644. 29321442 10.2169/internalmedicine.9860-17 PMC6028691

[12] Vincentelli A Susen S Le Tourneau T. Acquired von Willebrand syndrome in aortic stenosis. N Engl J Med 2003;349:343–349. 12878741 10.1056/NEJMoa022831

[13] Franchini M Makris M Santagostino E. Non-thrombotic-, non-inhibitor-associated adverse reactions to coagulation factor concentrates for treatment of patients with hemophilia and von Willebrand’s disease: a systematic review of prospective studies. Haemophilia 2012;18:e164–e172. 22250981 10.1111/j.1365-2516.2011.02745.x

[14] Galli-Tsinopoulou A Stylianou C Papaioannou G. Acquired von Willebrand’s syndrome resulting from untreated hypothyroidism in two prepubertal girls. Haemophilia 2006;12:687–689. 17083524 10.1111/j.1365-2516.2006.01355.x

[15] Lubinska M Swiatkowska-Stodulska R Kazimierska E. Acquired von Willebrand’s syndrome in a patient with severe primary hypothyroidism associated with myasthenia gravis in the course of autoimmune polyglandular syndrome type 3. Haemophilia 2007;13:675–676. 17880463 10.1111/j.1365-2516.2007.01488.x

[16] Berger J Schwartz J Ramachandran S. Review of von Willebrand disease and acquired von Willebrand syndrome for patients undergoing cardiac surgery. J Cardiothorac Vasc Anesth 2019;33:3446–3457. 31570241 10.1053/j.jvca.2019.08.025

[17] Feldmann C Zayat R Goetzenich A. Perioperative onset of acquired von Willebrand syndrome: Comparison between HVAD, HeartMate II and on-pump coronary bypass surgery. PLoS One 2017;12:e0171029. 28234916 10.1371/journal.pone.0171029 PMC5325196

[18] Icheva V Nowak-Machen M Budde U. Acquired von Willebrand syndrome in congenital heart disease surgery: results from an observational case-series. J Thromb Haemost 2018;16:2150–2158. 29908036 10.1111/jth.14208

[19] Solomon C Budde U Schneppenheim S. Acquired type 2A von Willebrand syndrome caused by aortic valve disease corrects during valve surgery. Br J Anaesth 2011;106:494–500. 21278152 10.1093/bja/aeq413

[20] Franchini M Mannucci PM. Acquired von Willebrand syndrome: focused for hematologists. Haematologica 2020;105:2032–2037. 32554559 10.3324/haematol.2020.255117 PMC7395262

[21] Peyvandi F Kouides P Turecek PL. Evolution of replacement therapy for von Willebrand disease: From plasma fraction to recombinant von Willebrand factor. Blood Rev 2019;38:100572. 31229334 10.1016/j.blre.2019.04.001

[22] Galletta E Galvanin F Bertomoro A. Acquired von Willebrand syndrome in patients with monoclonal gammopathy of undetermined significance investigated using a mechanistic approach. Blood Transfus 2023;21:74–82. 34694218 10.2450/2021.0121-21 PMC9918387

[23] Puronen CE Josephson NC Broudy VC. Acquired von Willebrand syndrome in a patient with monoclonal gammopathy of undetermined significance. Blood Coagul Fibrinolysis 2013;24:430–432. 23249617 10.1097/MBC.0b013e32835bfdde

[24] Saldanha A Veiga ME Okazaki E. Acquired von willebrand syndrome secondary to monoclonal gammopathy of undetermined significance: long-term remission after treatment with bortezomib. J Thromb Thrombolysis 2023;55:770–774. 37000318 10.1007/s11239-023-02799-6

[25] Biguzzi E Siboni SM le Cessie S. Increasing levels of von Willebrand factor and factor VIII with age in patients affected by von Willebrand disease: REPLY from original authors Biguzzi et al. J Thromb Haemost 2021;19:310. 33405387 10.1111/jth.15150

[26] Biguzzi E Siboni SM le Cessie S. Increasing levels of von Willebrand factor and factor VIII with age in patients affected by von Willebrand disease. J Thromb Haemost 2021;19:96–106. 32998182 10.1111/jth.15116

[27] Bitto N Tosetti G La Mura V. AB0, von Willebrand factor/factor VIII and portal vein thrombosis in decompensated cirrhosis: too late to unmask the culprit? Liver Int 2020;40:1788–1789. 32343027 10.1111/liv.14493

[28] Boucher AA Knutson S Young L. Prolonged elevations of factor VIII and von Willebrand factor antigen after multisystem inflammatory syndrome in children. J Pediatr Hematol Oncol 2023;45:e427–e432. 36730963 10.1097/MPH.0000000000002583 PMC10121725

[29] Bravo MI Raventos A Perez A. Non-additive effect on thrombin generation when a plasma-derived factor VIII/von Willebrand factor (FVIII/VWF) is combined with emicizumab in vitro. J Thromb Haemost 2020;18:1934–1939. 32379931 10.1111/jth.14887

[30] Murakawa M Okamura T Tsutsumi K. Acquired von Willebrand’s disease in association with essential thrombocythemia: regression following treatment. Acta Haematol 1992;87:83–87. 1585777 10.1159/000204725

[31] Jimenez-Yuste V Nunez L Alvarez-Roman MT. Efficacy and safety evaluation of Fanhdi((R)), a plasma-derived factor VIII/ von Willebrand factor concentrate, in Von Willebrand’s disease patients undergoing surgery or invasive procedures: a prospective clinical study. Haemophilia 2022;28:e23–e27. 34735040 10.1111/hae.14453 PMC10015987

[32] Mannucci PM Bettega D Cattaneo M. Patterns of development of tachyphylaxis in patients with haemophilia and von Willebrand disease after repeated doses of desmopressin (DDAVP). Br J Haematol 1992;82:87–93. 1419807 10.1111/j.1365-2141.1992.tb04598.x

[33] Castaman G Goodeve A Eikenboom J. Principles of care for the diagnosis and treatment of von Willebrand disease. Haematologica 2013;98:667–674. 23633542 10.3324/haematol.2012.077263 PMC3640108

[34] Goudemand J Bridey F Claeyssens S. Management of von Willebrand disease with a factor VIII-poor von Willebrand factor concentrate: Results from a prospective observational post-marketing study. J Thromb Haemost 2020;18:1922–1933. 32445594 10.1111/jth.14928 PMC7496521

